# Asymmetric fracture dislocation of the hips: case report

**DOI:** 10.11604/pamj.2024.47.179.43033

**Published:** 2024-04-09

**Authors:** Hassan Hachicha, Mohamed Chaker, Safouen Ben Brahim, Mehdi Meddeb, Hassen Makhlouf, Mondher Mestiri

**Affiliations:** 1 Adult Department, Mohamed Kassab Institute of Orthopedics, Mannouba, Tunisia

**Keywords:** Dislocation, hip, fracture, asymmetric, case report

## Abstract

Bilateral asymmetric hip fracture dislocation is an extremely rare entity. The injury is caused by a high velocity mechanism. We reported a case of bilateral hip fracture dislocation and its management. A 30-year-old man with no previous medical or surgical history was involved in a road accident involving a high-speed collision between two trucks. X-rays of the pelvis revealed asymmetrical bilateral fracture-luxation of the hips. The reduction of the hips was done under general anesthesia. Asymmetrical bilateral traumatic dislocation fracture of the hip is a rare serious injury. Reduction must be performed within 6 hours. Short- and long-term monitoring of the patient is essential.

## Introduction

Traumatic dislocation of the hip is a serious injury with a vital and functional prognosis in both the short and long term. The patient's vital and functional prognosis in the short and long term [1,2]. Traumatic hip dislocations account for 2-5% of all dislocations [3]. Bilateral forms are very rare. Their mechanism is quite specific and associated with road accidents. Bilateral hip dislocation is a rare injury, accounting for 1.25% of all hip dislocations [4]. Asymmetrical dislocations are even rarer, accounting for around 0.01-0.02% of all joint dislocations [4,5]. These patients must be considered as polytrauma patients, and the associated can be life-threatening. The basic treatment is to reduce dislocations within 6 hours to prevent avascular necrosis [4]. Our case illustrates this unusual lesion.

## Patient and observation

**Patient information:** a 30-year-old man with no previous medical or surgical history was involved in a road accident involving a high-speed collision between two trucks.

**Clinical findings:** the patient was hemodynamically and respiratorily stable. The primary investigation ruled out circulatory, respiratory and neurological problems. The position of the right lower limb was adducted and internally rotated. The position of the left lower limb was external rotation and abduction. The patient had no sensory-motor deficits and normal pulses in both lower limbs.

**Figure 1:**
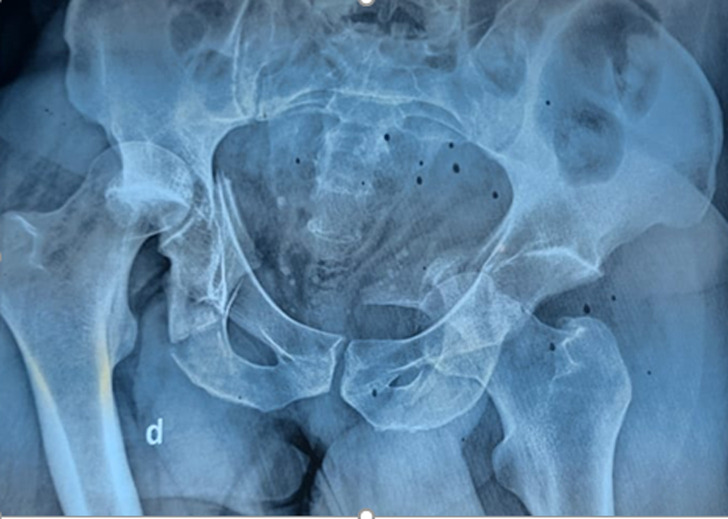
Asymmetric dislocated hip fracture

**Diagnostic assessment:** X-rays of the pelvis revealed asymmetrical bilateral fracture-luxations of the hips, with right superior-posterior and left anteroinferior dislocation (Figure 1).

**Figure 2:**
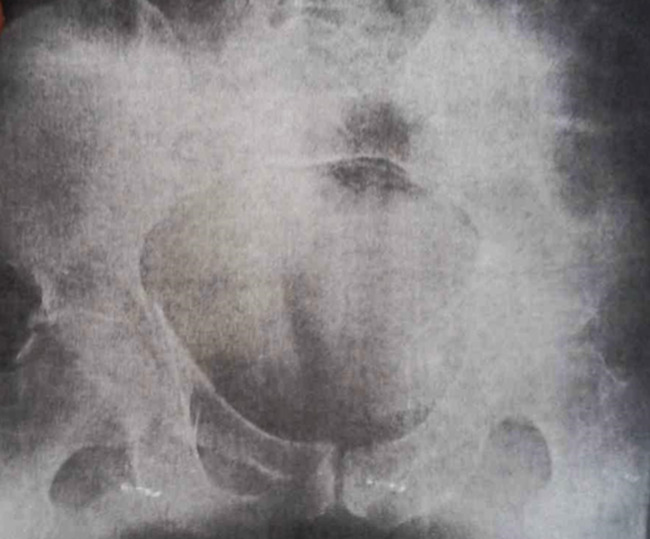
standard radiograph of the pelvis after reduction

**Figure 3:**
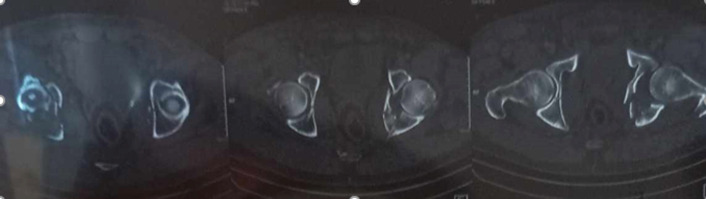
axial section of pelvis showing bilateral reduction of dislocations

**Therapeutic intervention:** the patient was admitted to the outpatient department and transferred within 6 hours to the operating room. Under general anaesthesia, a closed reduction was performed within 6 hours of trauma for both hip dislocations at the same time. Simultaneously: for the right hip dislocation, the reduction maneuver was performed on a hard surface, with counter-pressure on the anterosuperior iliac spines and traction in line with the femur, with the hip and knee flexed at 90 degrees; a clicking sound is audible during reduction. For the left hip, traction was applied along the axis of the deformity, flexing the hip to 90 degrees with an external rotation, with an audible click indicating reduction. After reduction, both hips were stable over the full range of motion distal neurovascular status. Post-reduction radiographs (Figure 2) and CT scans (Figure 3) demonstrated adequate of both hips. The patient was maintained in skin traction for both hips for 6 weeks.

**Patient's perspective:** the patient received analgesics and anti-thrombotic medication during his stay. He was discharged in the 3^rd^ week with the onset of soft callus construction.

**Informed consent:** the patient gives his informed consent.

## Discussion

Traumatic hip dislocation is more common in younger male patients younger age [4,6]. It accounts for 2-5% of all traumatic joint dislocations, and 90% are posterior [2,7]. Traumatic dislocation of the hip is symmetrical and bilateral (bilaterally anterior or bilaterally posterior) is a rare event, even more unusual is simultaneous asymmetrical (anterior and posterior) dislocation of both hips. This type of trauma usually occurs as a result of road accidents involving severe deceleration forces on a bent knee; associated injuries associated injuries are acetabular fractures and knee injuries [4,5]. The most common example of a bilateral hip dislocation is that of the car driver not wearing a seatbelt, or in front-seat passengers not unrestrained front-seat passengers [4,8]. During the rapid deceleration of the vehicle, the body pivots forward on the fixed legs and the knees strike the dashboard, transmitting the dislocating force to the hip joints [8]. Depending on the position of the legs at the moment of impact, the impact may cause anterior or posterior dislocation [4,9,10].

The 1^st^-line complementary examination that should be requested and that highlights these lesions is a frontal X-ray of the pelvis. Associated lesions include acetabular fracture, femoral fracture (4% of patients), knee ligament damage or fracture of the patella or proximal tibia (25%), damage to the femoral artery, venous thrombosis and sciatic nerve injury (7-19%) [2,5]. Computed tomography is a good diagnostic method and provides additional information, but should never delay urgent management treatment, which is limited to reducing the dislocation. The reduction of these traumatic hip dislocations must be performed within a six hours post-trauma according to Hougaard [11] to twelve hours post-trauma, whether open or closed, to reduce the risk of complications [6,12]. Ideally, reduction should always be performed under general anaesthetic. It is performed on a curarized patient, using gentle, non-aggressive maneuvers [13]. Reduction maneuvers vary according to the type of hip dislocation.

For posterior dislocations, the Boehler maneuver [14] is performed in the decubitus on a hard surface, with counter-pressure on the anterosuperior iliac spines. It consists of a traction movement in the axis of the femur, with the hip and knee in 90-degree flexion audible during reduction [13,14].

For anterior dislocations, the Allis maneuver is performed [13,14], with a traction in the axis of the deformity, with the hip flexed to 90 degrees. This may be combined with small movements of internal and external rotation. Abduction movements should be avoided, as they are associated with a high risk of fracture of the hip neck, according to Bigelow [14].

Complications are manifold and include avascular necrosis of the femoral head, post-traumatic arthritis and sciatic nerve injury [2,6]. Other potential complications reported in the literature include heterotopic ossifications, deep-vein thrombosis and limitation of hip movement [2]. The rates of these complications vary according to the type of dislocation and whether open or closed reduction is performed. Delayed management of these dislocations leads to a significant increase complications, namely avascular necrosis of the femoral head and post-traumatic arthritis [15]. Neurovascular lesions may accompany hip dislocations in which the sciatic nerve is most often injured [2,15]. The case illustrated represents an unusual, uncommon and severe combination of injuries resulting from a high-speed vehicle accident. Hip dislocation is an orthopaedic emergency that needs to be treated in good time without forgetting that the patient's vital prognosis is always the overriding paramount concern.

## Conclusion

Asymmetrical bilateral traumatic dislocation fracture of the hip is a rare serious injury. Reduction must be performed within 6 hours in order to optimize the prognosis of this condition. Short- and long-term monitoring of the patient is essential, as long-term arthrosic is unavoidable.

## References

[1] Jean-Eric KK, Blaise YL, Régis AAJ, Yannick BG, Michel K (2021). Luxation traumatique bilatérale asymétrique de la hanche chez un adulte.

[2] Sahin O, Ozturk C, Dereboy F, Karaeminogullari O (2007). Asymmetrical bilateral traumatic hip dislocation in an adult with bilateral acetabular fracture. Arch Orthop Trauma Surg.

[3] Elouakili I, Chahbouni M, Najib A, Rhanim A, Kharmaz M, Lamrani MO (2011). Luxation traumatique bilatérale de la hanche. J Traumatol Sport.

[4] Alshammari A, Alanazi B, Almogbil I, Alfayez SM (2018). Asymmetric bilateral traumatic hip dislocation: A case report. Ann Med Surg.

[5] Buckwalter J, Westerlind B, Karam M (2015). Asymmetric Bilateral Hip Dislocations: a Case Report and Historical Review of the Literature. Iowa Orthop J.

[6] Alnaser AAMA, Abd-Elmaged HMA, Mohammed FEA, Abd ALLAH RAAA, Mohamed Ahmed Hussien MA (2022). A bilateral asymmetrical hip dislocation: A rare case report. Clin Case Rep.

[7] Lam F, Walczak J, Franklin A (2001). Traumatic asymmetrical bilateral hip dislocation in an adult. Emerg Med J EMJ.

[8] Loupasis G, Morris EW (1998). Asymmetric bilateral traumatic hip dislocation. Arch Orthop Trauma Surg.

[9] Shukla PC, Cooke SE, Pollack CV, Kolb JC (1993). Simultaneous asymmetric bilateral traumatic hip dislocation. Ann Emerg Med.

[10] Gittins ME, Serif LW (1991). Bilateral traumatic anterior/posterior dislocations of the hip joints: case report. J Trauma.

[11] Hougaard K, Thomsen PB (1987). Coxarthrosis following traumatic posterior dislocation of the hip. J Bone Joint Surg Am.

[12] Braun ME, Loose O, Schmittenbecher P, Schneidmüller D, Strüwind C, Schwerk P (2023). Epidemiology and injury morphology of traumatic hip dislocations in children and adolescents in Germany: a multi-centre study. Eur J Trauma Emerg Surg Off Publ Eur Trauma Soc.

[13] Akiki A, Duvoisin C, Krupp F, Kombot C (2012). Luxations du membre inférieur: les reconnaître et les traiter. Rev Med Suisse.

[14] Burdin G, Hulet C, Slimani S, Coudane H, Vielpeau C (2004). Luxations traumatiques de hanche: luxations pures et fractures de tête fémorale. EMC-Rhumatol Orthopédie.

[15] Epstein NE (2016). Older literature review of increased risk of adjacent segment degeneration with instrumented lumbar fusions. Surg Neurol Int.

